# Automatic effects of covert practice

**DOI:** 10.1177/17470218211007138

**Published:** 2021-04-05

**Authors:** Baptist Liefooghe, Ariane Jim, Jan De Houwer

**Affiliations:** 1 Department of Psychology, Utrecht University, Utrecht, The Netherlands; 2 Department of Experimental Clinical and Health Psychology, Ghent University, Gent, Belgium

**Keywords:** Learning, automaticity, covert practice

## Abstract

Automatic behaviour is supposedly underlain by the unintentional retrieval of processing episodes, which are stored during the repeated overt practice of a task or activity. In the present study, we investigated whether covertly practicing a task (e.g., repeatedly imagining responding to a stimulus) also leads to the storage of processing episodes and thus to automatic behaviour. Participants first either responded overtly or covertly to stimuli according to a first categorization task in a practice phase. We then measured the presence of automatic response-congruency effects in a subsequent test phase that involved a different categorization task but the same stimuli and responses. Our results indicate that covert practice can lead to a response-congruency effect. We conclude that covert practice can lead to automatic behaviour and discuss the different components of covert practice, such as motor imagery, visual imagery, and inner speech, that contribute to the formation of processing episodes in memory.

Covert practice consists of the mental or internal rehearsal of an activity with the aim of improving performance in this activity (e.g., Jackson et al., 2003). An activity can be rehearsed in different ways and covert practice encompasses different processing components. Foremost covert practice is assumed to involve the covert simulation of the motor components of an activity (i.e., motor imagery; Decety & Grèzes, 1999; Guillot & Collet, 2010; Moran et al., 2012). This involves the activation of motor representations, which are also involved in the preparation and initiation of actual movements (Jeannerod, 1994, 2001, 2004; see also Burianová et al., 2013; Hardwick et al., 2018; Hétu et al., 2013; Kraeutner et al., 2014). Covert practice, however, is also underlain by inner speech, which involves the covert repetition of verbal labels attached to different elements of an activity (e.g., “grasp,” “move left,” etc.. . .; e.g., Hall et al., 1997). Finally, covert practice calls upon visual imagery, as it involves the representation of not only actions but also the stimulus or object on which these actions need to be performed (e.g., a mental image of a moving tennis ball when covertly practicing a backhand, see also Denis, 1985; Fery, 2003; Ingram et al., 2016).

It has frequently been asserted that covertly practicing a task optimises performance in a variety of domains such as in athletics (see Weinberg, 2008, for a review), typing (Nyberg et al., 2006), music (e.g., Highben & Palmer, 2004; Lim & Lippman, 1991), or surgery (e.g., Rogers, 2006). These examples suggest that covert practice may be helpful in automating behaviour as it is the case for overt practice. Automatic behaviour is considered to be skillful, quick, effortless and unintentional (see Bargh, 1992; Moors, 2016; Moors & De Houwer, 2006, for elaborate discussions). A common assumption is that automatic behaviour develops by repeatedly overtly performing an action or task (i.e., practice; e.g., Logan, 1988; Shiffrin & Schneider, 1977). Although different views exist on the processes underlying automatic behaviour (e.g., Anderson, 1992; Tzelgov et al., 2000), an influential account relating automatic behaviour to practice was introduced by Logan (1988), who conceived automatic behaviour as the result of the direct retrieval of processing episodes from long-term memory. Whereas initial task performance is guided by a more controlled processing route, each application of a task results in the storage of a processing episode in memory. Such an episode represents a multitude of information, such as the relevant stimulus, the response or action performed on that stimulus, and the task or context in which this was done. Importantly, when the relevant stimulus is presented again later in time, this processing episode is automatically retrieved from memory, thus prompting responding. When frequently applying or practicing a task, an increasing number of episodes are stored in memory, which in turn are retrieved at a later stage. Logan (1988) assumes that automatic behaviour is attained when task performance is completely determined by episodic retrieval and no longer based on the controlled processing route (see also Logan, 1992, 2002; Schmidt et al., 2016).

Theeuwes et al. (2018) reported experimental evidence suggesting that covert practice may also lead to the storage of processing episodes. These authors measured the beneficial effect of overtly or covertly practicing new instructions, which related a stimulus (e.g., “rabbit”) to a sequence of three responses (e.g., pressing the letter-keys “s,” “p,” “r”). Overt practice consisted of applying these instructions a number of times. Covert practice required participants to imagine applying the instructions, without actually doing so. In a test phase, the effect of overt and covert practice was compared with a control condition in which the instructions could not be practised. Overt practice led to better test performance compared with covert practice. However, covert practice also had a small beneficial effect, both in terms of speed and accuracy, compared with the control condition. In line with Logan’s (1988) account, it could be argued that processing episodes were not only stored during overt practice, but also during covert practice. These episodes then improved test performance on the basis of automatic retrieval. However, automatic behaviour is indexed not only in terms of speed, or skilfulness in general, but also by the extent that a behaviour is performed unintentionally when being irrelevant (e.g., Moors & De Houwer, 2006). In the present study, we investigated whether covert practice can also lead to unintentional behaviour by testing whether covert practice can induce response-congruency effects.

A robust observation is that overtly applying stimulus-response mappings leads to response-congruency effects. This is typically investigated by using an item-specific priming paradigm (e.g., Moutsopoulou et al., 2015; Pfeuffer et al., 2017; Shiffrin & Schneider, 1977; Verbruggen & Logan, 2008; Waszak et al., 2003). Participants, for instance, first categorise stimuli by pressing a left or a right key during a practice phase in which the response mappings remain unchanged (e.g., non-mechanical => left vs. mechanical => right). In a subsequent test phase, stimuli of the practice phase are again presented but the categorization rule is different and kept constant (e.g., small => left, large => right). Stimuli can be *congruent* and require the same response in both phases (e.g., a small non-mechanical object such as a pawn) or they are *incongruent* and require different responses (e.g., a large non-mechanical object, such as a gown). Typically, reaction times are longer and error rates higher on incongruent stimuli compared with congruent stimuli (i.e., a response-congruency effect), which is considered an index of the unintentional retrieval of processing episodes stored during the practice phase. For instance, when performing the non-mechanical versus mechanical task (non-mechanical => left vs. mechanical => right) in the practice phase, the stimulus “pawn” is responded to overtly by pressing a left key. This leads to the storage of an episode including an association between the stimulus “pawn” and the response “left.” When “pawn” is presented in the test phase, which involves the size task (small => left, large => right), the association between “pawn” and “left” is automatically retrieved. The stimulus “pawn” is congruent and only one response (“left”) is retrieved. However, when considering the stimulus “gown” in the context of the same example, “gown” becomes associated with a left response in the practice phase, but necessitates a right response in the test phase. When responding to “gown” in the test phase (i.e., pressing right), the competing response is thus retrieved (i.e., left). As a result, response performance is impaired (i.e., longer reaction times, higher error rates) on incongruent compared with congruent stimuli.

In the present study, we adapted the item-specific priming paradigm to test whether performing the practice phase covertly (i.e., imagining responding without actually doing so) also leads to response-congruency effects in the subsequent test phase, a finding that would indicate that covert practice can also lead to the storage of processing episodes in memory. To see whether our procedure was adequate to observe practice-based response-congruency effects in the first place, we also included a condition in which participants overtly responded to the stimuli in the practice phase. In addition, we were concerned that response-congruency effects in the test phase could also be induced by the mere instruction of two sets of category-response rules (small vs. large; mechanical vs. non-mechanical), without stimuli being overtly or covertly responded to in the practice phase (see Longman et al., 2019). To control for this, congruent and incongruent stimuli were included in the test phase, which were not presented in the practice phase and thus not exposed to any form of practice.

Besides testing whether covert practice leads to response-congruency effects, we also investigated the extent to which motor imagery was involved during covert practice. Mental-chronometry procedures have repeatedly demonstrated that the time needed to perform a particular action covertly co-varies with the time needed to execute an action overtly (i.e., isochrony, see Guillot et al., 2012; McAvinue & Robertson, 2008, for reviews). For instance, Decety et al. (1989) observed that increasing the length of a particular walking distance increases not only the actual walking time but also the imagined walking time, which offers an indication that kinesthetic information is included during the imagery experience. Accordingly, we manipulated the physical distance participants had to move their response hand and measured whether this affected the time to imagine responding to a stimulus (see Theeuwes et al., 2018, for a similar approach). Our prediction was that if kinesthetic information is represented during covert practice, which points towards the involvement of motor imagery, then this estimated time should be longer when the response requires to cover a larger physical distance.

## Method

Software, stimulus materials, raw data, and the corresponding processing scripts are available at https://osf.io/9xhde.

### Participants

Sixty-four students (*M_age_* = 21.70; *SD_age_* = 4.35; 53 female; 56 right-handed) at Ghent University participated in return for a 10 Euro payment. Participants were right-handed and naive to the purpose of the experiment. They were randomly assigned to one of the two practice conditions (overt practice: *n* = 32; covert practice: *n* = 32). With 32 participants per condition, we had a power of .80 for detecting a medium-sized effect within each condition. Ethical approval for this project was granted by the Ethical Committee of the Faculty of Psychology and Educational Sciences of Ghent University.

### Tasks and materials

**Figure 1.**
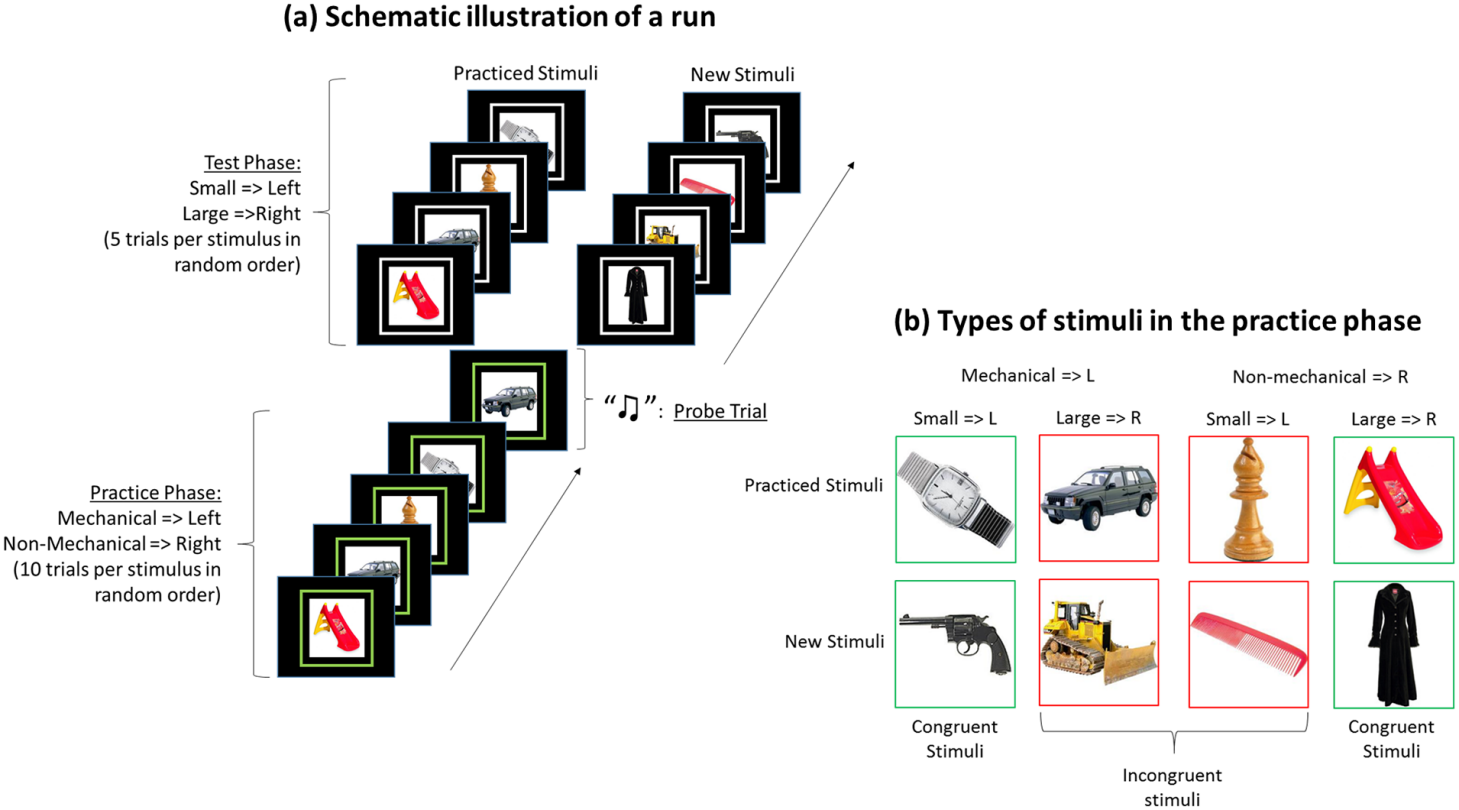
Schematic example of a block consisting of a practice phase and a test phase (Panel a); types of stimuli in the practice phase of that specific block (Panel b).

Participants were presented with different blocks of trials, each consisting of a practice phase and a test phase (see Figure 1, Panel a). Both phases included a different categorization task, namely non-mechanical versus mechanical or small versus large (compared with a basketball). In the overt-practice condition, 17 participants received the non-mechanical versus mechanical task in the practice phase and 15 participants the small versus large task. In the covert-practice condition, 16 participants received the mechanical versus non-mechanical task in the practice phase and 16 participants the small versus large task.

Each task required either a left or a right response (e.g., mechanical => left, non-mechanical => right; small => left, large => right). The practice phase included a first categorization task (e.g., mechanical => left, non-mechanical => right) during which four types of stimuli were each presented 10 times in a random order. These stimuli belonged to four categories: small non-mechanical objects (e.g., a pawn), small mechanical objects (e.g., a watch), large non-mechanical objects (e.g., a gown), or large mechanical objects (e.g., a car). Participants were instructed to practice this first categorization task, either overtly (i.e., overtly responding to the stimuli) or covertly (i.e., imagining to respond to the stimuli) on these stimuli.

At the end of the practice phase, a single probe trial was presented during which all participants had to respond overtly—following the categorization rule of the practice phase—to one of the stimuli that were practised (e.g., car => left). This probe trial served two purposes. First, it was included to motivate participants to genuinely perform covert responses during the covert practice phase (i.e., it offered reason of practicing). Second, it allowed us to compare the practice benefit of the covert-practice phase and the overt-practice phase. Following the probe trial, the test phase started. In the test phase, the second categorization task was now relevant (e.g., small => left, large => right), but the stimuli of the practice phase were reused and stimuli either required the same response as in the practice phase (i.e., congruent stimulus) or a different response (i.e., incongruent stimulus).

Four new stimuli were added to the test phase, which were not presented in the practice phase. Each new stimulus belonged to one of the four stimulus categories (small non-mechanical objects; small mechanical objects; large non-mechanical objects; or large mechanical objects). Accordingly, the new stimuli could also be congruent or incongruent. For instance, consider a block with the following two sets of categorization rules: practice-phase: mechanical => left, non-mechanical => right; test-phase: small => left, large => right. Even if a stimulus is only presented in the test phase, it can be congruent across both sets of rules (e.g., a watch) or incongruent (e.g., a plane).

A total of 72 stimulus pictures were selected from the picture-set created by Brady et al. (2008) and Moutsopoulou et al. (2015). This selected set consisted of small non-mechanical objects, large non-mechanical objects, small mechanical objects, and large mechanical objects. Per category 18 pictures were selected. Nine blocks of trials were created on the basis of these stimuli, by assigning two stimuli of each category to each block. Each block thus used eight new stimuli. Within each block, four stimuli (one per category) were used both in the practice and the test phase. The remaining four stimuli (one per category) were only used in the test phase.

The two categorization tasks used the same response-keys, namely the “X” (i.e., left) and the “O” letter-key (i.e., right) on an AZERTY keyboard. Relative to the spacebar, which functioned as the rest-key, the “X” is physically close and the “O” is physically more distant. The category-to-response mappings of both tasks varied randomly across participants. Stimuli were pictures, which were presented in the centre of a black screen and surrounded by a square. The colour of the square indicated which task participants had to apply. The practice phase was cued by a green square. The test phase was indicated by a white square. At the end of the practice phase, all participants overtly responded to a single probe trial of the practice phase. The onset of this probe trial was indicated by a 440 hertz tone.

### Procedure

The experiment was programmed in OpenSesame (Mathôt et al., 2012). Participants were tested individually. After signing an informed consent, the overall instructions of the experiment were provided on screen and paraphrased. To encourage participants to engage in motor imagery in the covert-practice condition during the whole period of the experiment, an electrode was connected to their right index finger, which was attached to a bogus device (see also Scheil et al., 2020; Scheil & Liefooghe, 2018; Theeuwes et al., 2018). Participants in the covert-practice condition were told that we were able to measure the degree to which they performed motor imagery. The electrode was also applied in the overt-practice condition and participants were told that we measured motor-activity in their response hand. In both conditions the experimenter was continuously present during the experiment to monitor participants’ performance from behind a desktop connected to the bogus device, which was a defected voice-key amplifier. The experimenter also corrected participants if they did move their hand explicitly towards one of the response-keys in the covert-practice condition.

The experiment consisted of nine blocks (one exercise block and eight test blocks). A schematic outline of a block is presented in Figure 1. Each block used eight new stimuli and started with the practice phase in which 4 stimuli (one stimulus per stimulus category) were used. The practice phase consisted of 40 trials during which each stimulus was practised for 10 trials in a random order. Accordingly, we had 320 practice trials (8 test blocks × 40 practice trials per block).

On each trial of the covert-practice condition, participants were first required to hold down the spacebar (rest-key), a fixation cross then appeared for 500 ms. Following this fixation cross, the stimulus appeared in the centre of the screen. Participants were required to release the spacebar, imagine responding to the stimulus, and press the spacebar down again. Participants had a response limit of 4,000 ms to do so.

In the overt-practice condition, the same sequence of actions was required, but participants needed to actually press the left or right response-key. Participants had 2,000 ms to release the spacebar starting from the stimulus onset and another 2,000 ms to press the response-key. No response deadline was imposed for returning to the spacebar. To equate both practice conditions, no feedback was provided with respect to the correctness of the responses made in the overt-practice condition.

Following the practice phase, the probe trial was presented in which participants had to apply the categorization rule of the practice phase. One of the four practised stimuli was randomly selected and all participants had to respond overtly to this probe by using the sequence of actions used in the overt-practice condition (i.e., release spacebar, press left or right key, and return to the spacebar). Similarly, all participants had to respond overtly to the stimuli in the test phase by releasing the spacebar, entering the correct response-key, and returning to the spacebar. In the test phase, eight stimuli were presented: the four stimuli that were used in the practice phase and four new stimuli (1 per stimulus category). Each stimulus was presented 5 times and the test phase thus consisted of 40 trials. In total, we had 90 observations for each cell of the orthogonal combination of Stimulus Congruency and Stimulus Type (practised, new). As in the practice phases, the inter-trial interval was 500 ms. In addition, response feedback was now presented by displaying the messages “correct” or “fout” (wrong in Dutch) for 750 ms. At the end of a block, a break was inserted and participants were presented with feedback on their average response speed and accuracy. Participants started the next block by pressing down the spacebar. The experiment lasted for approximately 50 min.

## Results

All data processing and analyses were performed by using R (R Core Team, 2017). Analyses of variance (ANOVAs) were calculated by using “afex” and follow-up contrasts on the model estimates by using “phia” (De Rosario-Martinez, 2015). We first consider the results of the test phase. Next, we present the practice phase.

### Test phase

A potential concern of our design is that in each mini block one of the practised stimuli in the test phase also served as a probe stimulus in the preceding practice phase. It could be argued that the response-congruency effect observed for the practised stimuli in the covert-practice condition was not uniquely based on covert practice, but was also induced by overtly responding to the stimulus that served as the probe at the end of the practice phase. We therefore excluded the stimuli that served as a probe from the analyses of the test phase.

#### Reaction times

**Figure 2.**
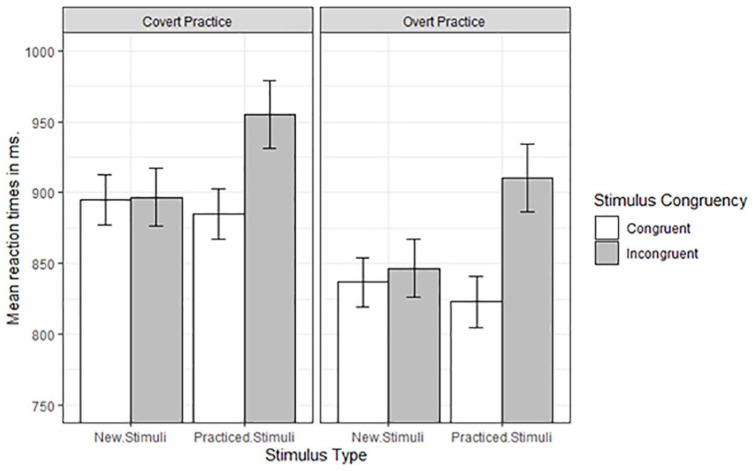
Mean reaction times of the test phase in milliseconds as a function of Condition, Stimulus Type, and Stimulus Congruency. Error bars denote standard errors.

Reaction times (RTs) were defined as the time between the stimulus onset and the left-right response to the stimulus. Only RTs of correct trials in test phases, which followed a correct response to the probe of the practice phase, were considered. This led to the removal of 8.33% of the trials. Next, for each participant RTs exceeding 2.5 standard deviation of each cell mean were considered as outliers. This led to the removal of 3.09% of the trials. RTs were subjected to a 2 (Practice Condition) by 2 (Stimulus Congruency) by 2 (Stimulus Type: practised, new) mixed ANOVA with repeated measures on the last two factors. Cell means and corresponding standard errors are presented in Figure 2.

The difference in RTs following covert practice (*M* = 907 ms, *SE* = 19) and overt practice (*M* = 854 ms, *SE* = 19) did not reach statistical significance, *F*(1,62) = 3.86, *MSe* = 47718, *p* = .05, $\eta_{p}^{2}$ = .06. RTs were faster on congruent stimuli (*M* = 860 ms, *SE* = 12) compared with incongruent stimuli (*M* = 902 ms, *SE* = 15), *F*(1,62) = 58.15, *MSe* = 1968, *p* < .001, $\eta_{p}^{2}$= .48. RTs were slower on practised stimuli (*M* = 893 ms, *SE* = 14) compared with new stimuli (*M* = 869 ms, *SE* = 13), *F*(1,62) = 34.44, *MSe* = 1136, *p* < .001, $\eta_{p}^{2}$= .36.

The two-way interaction between Stimulus Congruency and Stimulus Type (practised, new) was significant, *F*(1,62) = 98.20, *MSe* = 868, *p* < .001, $\eta_{p}^{2}$= .61. For the practised stimuli, a response-congruency effect was present: RTs were shorter on congruent stimuli (*M* = 854 ms, *SE* = 13) than on incongruent stimuli (*M* = 932 ms, *SE* = 17), *F*(1,62) = 97.82, *p* < .001, $\eta_{p}^{2}$= .61. For the new stimuli, the response-congruency effect (congruent stimuli: *M* = 866 ms, *SE* = 12; incongruent stimuli: *M* = 871 ms, *SE* = 15) was not significant, *F*(1,62) = 1.33, *p* = .25, $\eta_{p}^{2}$= .02.

The two-way interaction between Practice Condition and Stimulus Type was not significant, *F* < 1. The main effect of Stimulus Type did not differ reliably between both practice conditions. The three-way interaction was also not significant, *F* < 1. As can be seen in Figure 2, we observed a response-congruency effect for the practised stimuli in the covert-practice condition, *F*(1,62) = 38.88, *p* < .001, $\eta_{p}^{2}$= .39, as well as a response-congruency effect for the practised stimuli in the overt-practice condition, *F*(1,62) = 60.09, *p* < .001, $\eta_{p}^{2}$= .49.

Two components can contribute to the response-congruency effect. The unintentional retrieval of a competing response from memory, which leads to a performance cost on incongruent trials and/or facilitative effects on congruent trials. Whereas facilitative effects on congruent trials could be mediated strategically (i.e., intentional control), interference effects on incongruent trials offer a more conclusive case for the hypothesis that covert practice leads to unintentional retrieval, which impedes behaviour. Accordingly, we compared practised congruent stimuli with new congruent stimuli, on one hand, and practised incongruent stimuli with new incongruent stimuli, on the other hand. In the covert-practice condition, the difference between congruent practised stimuli (884 ms) and congruent new stimuli (895 ms) was not significant, *F*(1,62) = 2.89, *p* = .09, $\eta_{p}^{2}$= .04. The difference between incongruent practised stimuli (955 ms) and incongruent new stimuli (896 ms) was significant, *F*(1,62) = 37.22, *p* < .001, $\eta_{p}^{2}$= .28. For the overt-practice condition, the difference between congruent practised stimuli (823 ms) and congruent new stimuli (837 ms) was significant, *F*(1,62) = 5.70, *p* < .05, $\eta_{p}^{2}$= .08. The difference between incongruent practised stimuli (910 ms) and incongruent new stimuli (846 ms) was also significant, *F*(1,62) = 44.32, *p* < .001, $\eta_{p}^{2}$= .42. The findings thus indicate the presence of performance costs on incongruent trials.

**Figure 3.**
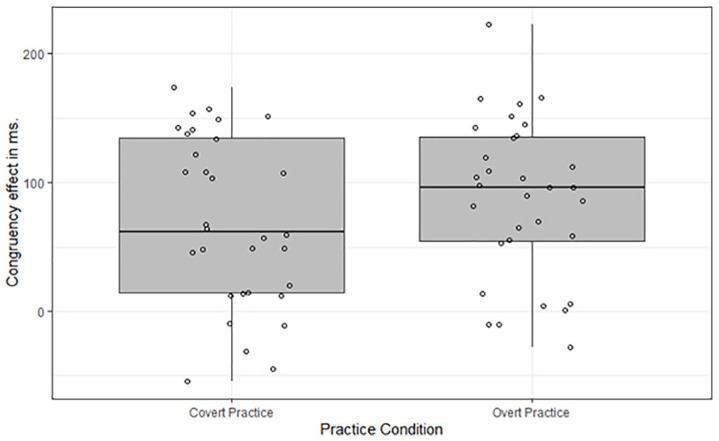
Boxplot of the response-congruency effects in milliseconds (Incongruent—Congruent) in the test phase per participant in the overt-practice and covert-practice condition. Circles denote participants.

Because the extent to which participants engage in covert practice is subject to inter-individual differences (e.g., Hall, 1985), the response-congruency effect observed in the covert-practice condition may have been driven by a subset of the participants in that condition. Accordingly, for both practice conditions we calculated the response-congruency effect per participant and created boxplots on the basis of these difference scores to detect participants with extreme response-congruency effects (i.e., outliers). The boxplots are presented in Figure 3 and offer a graphical representation of the sample distribution for both response-congruency effects. The middle line indicates the median, the hinges the first and third quartile. Outliers are detected by first calculating the difference between the first (Q1) and the third (Q3) quartile (i.e., inter-quartile range [IQR]). Values 1.5 times the IQR below Q1 and 1.5 times the IQR above Q3 are considered as outliers (Tukey, 1977). The whiskers relate Q1 and Q3 to the minimum and maximum value, respectively, which do not fall outside the IQR. As can be seen in Figure 3, no outliers were detected, neither in the covert-practice condition nor in the overt-practice condition.

#### Error rates

**Figure 4.**
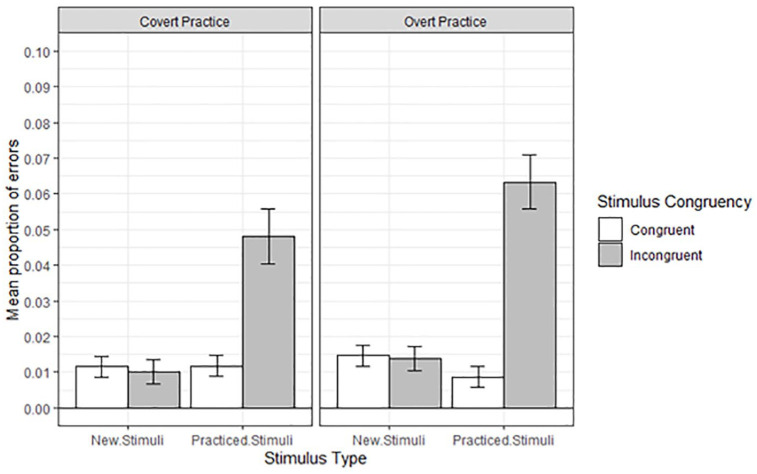
Mean proportion of errors in the test phase, as a function of Condition, Stimulus Type, and Stimulus Congruency. Error bars denote standard errors.

Error rates were also subjected to a 2 (Practice Condition) by 2 (Stimulus Congruency) by 2 (Stimulus Type: practised, new) mixed ANOVA with repeated measures on the last two factors. Cell means and corresponding standard errors are presented in Figure 4. The difference in error rates following overt practice (*M* = .0251, *SE* = .0026) and covert practice (*M* = .0203, *SE* = .0026) did not reach statistical significance, *F*(1,62) = 1.68, *MSe* = .0009, *p* = .20, $\eta_{p}^{2}$= .03. Less errors were made to congruent stimuli (*M* = .0116, *SE* = .0017) compared with incongruent stimuli (*M* = .0338, *SE* = .0032), *F*(1,62) = 36.31, *MSe* = .0009, *p* < .001, $\eta_{p}^{2}$= .37. More errors were made on practised stimuli (*M* = .0329, *SE* = .0029) compared with new stimuli (*M* = .0126, *SE* = .0016), *F*(1,62) = 49.55, *MSe* = .0005, *p* < .001, $\eta_{p}^{2}$= .44.

The two-way interaction between Stimulus Congruency and Stimulus Type was significant, *F*(1,62) = 71.64, *MSe* = .0005, *p* < .001, $\eta_{p}^{2}$= .54. For the practised stimuli, a response-congruency effect was present: Error rates were lower on congruent stimuli (*M* = .0102, *SE* = .0021) than on incongruent stimuli (*M* = .0557, *SE* = .0053), *F*(1,62) = 64.10, *p* < .001, $\eta_{p}^{2}$= .51. For the new stimuli, the response-congruency effect (congruent stimuli: *M* = .0131, *SE* = .0021; incongruent stimuli: *M* = .0120, *SE* = .0024) was not significant, *F* < 1.

**Figure 5.**
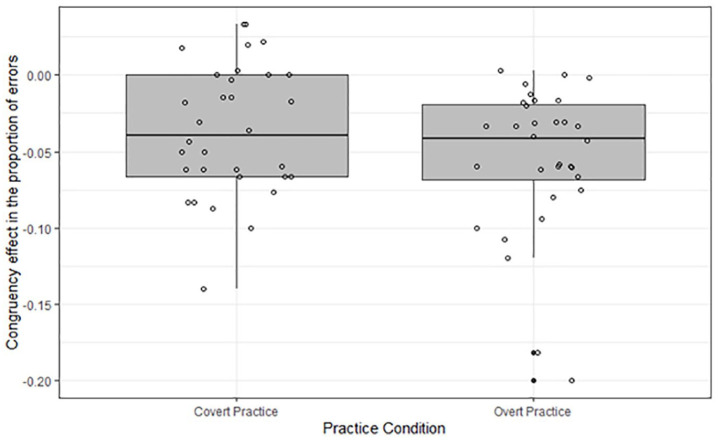
Boxplot of the response-congruency effects in the proportion of errors (Incongruent – Congruent) in the test phase per participant in the overt-practice and covert-practice condition. Circles denote participants.

The two-way interaction between Practice Condition and Stimulus Type, *F* < 1, the two-way interaction between Practice Condition and Stimulus Congruency, *F*(1,62) = 1.68, *MSe* = .0009, *p* = .19, $\eta_{p}^{2}$= .03, as well as the three-way interaction, *F*(1,62) = 2.54, *MSe* = .0005, *p* = .12, $\eta_{p}^{2}$= .04, were not significant. As can be seen in Figure 4, a response-congruency effect was present on the practised stimuli in the covert-practice condition, *F*(1,62) = 46.27, *p* < .001, $\eta_{p}^{2}$= .43, and in the overt-practice condition, *F*(1,62) = 20.43, *p* < .001, $\eta_{p}^{2}$= .25. As for the response times, we constructed boxplots on the basis of participants’ mean response-congruency effects. As can be seen in Figure 5, we observed two outliers in the overt-practice condition, which had very sizable mean response-congruency effects. In the covert-practice condition, no outliers were observed.

### Practice phase

The common dependent variable in both practice conditions was the interval between releasing and entering the spacebar (inter-spacebar-interval). In the overt-practice condition, we detected 2.86% of errors. These trials were not discarded from the analysis. This way, a more proper comparison was possible with the covert-practice condition in which no accuracy data were available and we could thus not estimate the contribution of incorrectly estimated responses to the inter-spacebar intervals.

Per participants and per cell of the design, inter-spacebar intervals were trimmed for outliers and 2.23% of the observations were removed. To see whether covert and overt responses during the practice phase were modulated by the physical distance between the response-keys and the spacebar, mean inter-spacebar-intervals were subjected to a 2 (Practice Condition) by 2 (Response-Key Distance: short vs. long) mixed ANOVA with repeated measures on the last factor.

**Figure 6.**
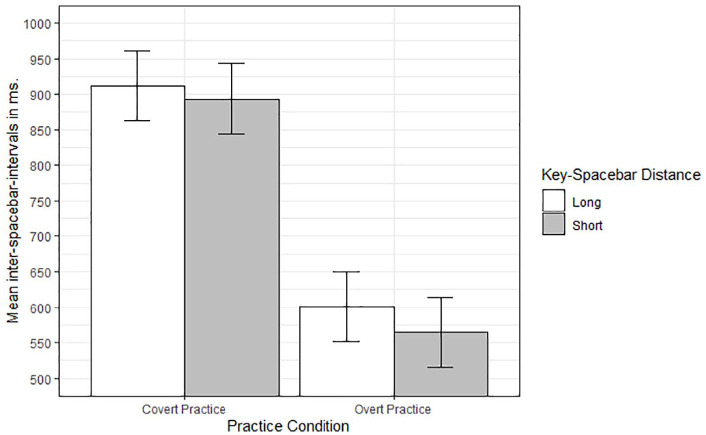
Mean inter-spacebar-intervals in milliseconds in the practice phase as a function of Condition and Key-Spacebar Distance. Error bars denote standard errors.

Inter-spacebar intervals were significantly longer in the covert-practice condition (*M* = 903 ms, *SE* = 49) compared with the overt-practice condition (*M* = 582 ms, *SE* = 49), *F*(1,62) = 21.33, *MSe* = 153740, *p* < .001, $\eta_{p}^{2}$ = .26. The main effect of response-key distance was also significant, *F*(1,62) = 12.85, *MSe* = 1899, *p* < .001, $\eta_{p}^{2}$ = .17. Inter-spacebar intervals were 28 ms longer when the response-key distance was large compared with the release-key (i.e., the “O” compared with the spacebar) than when this distance was smaller (i.e., the “X” compared with the spacebar). The two-way interaction was not significant, *F*(1,62) = 1.40, *MSe* = 1899, *p* > .24, $\eta_{p}^{2}$ = .02. However, as can be seen in Figure 6, the effect of distance was more pronounced in the overt-practice condition (36 ms), *F*(1,62) = 11.36, *p* < .01, $\eta_{p}^{2}$ = .15, compared with the covert-practice condition (18 ms), *F*(1,62) = 2.89, *p* = .09, $\eta_{p}^{2}$ = .05.

**Figure 7.**
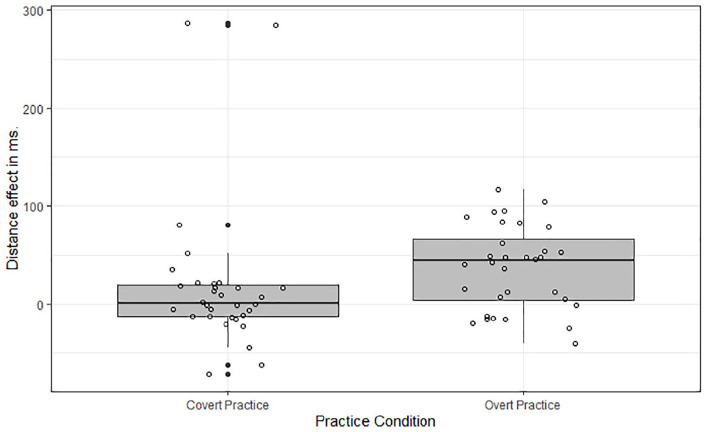
Boxplot of the key-spacebar distance effects (Long—Short) in the practice phase, per participant in the overt-practice and covert-practice condition. Circles denote participants in each condition. Black circles indicate outliers.

In line with our previous analyses, we investigated whether this interaction was driven by participants with extreme difference scores. Per participant the difference in inter-spacebar-intervals between both distances was calculated and boxplots were created (Figure 7). In the covert-practice condition, five participants were detected as outliers. When removing these outliers in a follow-up contrast the distance effect was no longer significant in the covert-practice condition, *F* < 1.

### Correlation between practice and test phase

To assess the extent to which participants engaged in motor imagery during covert practice, we calculated the mean inter-spacebar-interval in the practice phase and the test phase per participant and correlated both per practice condition. As such, we could investigate how strongly the time needed to imagine responding correlates with the actual time needed to perform a response, which offers an indication of motor imagery (i.e., isochrony, see Guillot et al., 2012 for a review). For the covert-practice condition, the Pearson product-moment correlation was not significant: *r* = .23, *p* = .22. In contrast, in the overt-practice condition this correlation was significant: *r* = .95, *p* < .001.

In view of the length of the practice phase (40 trials), it was possible that participants genuinely started with motor imagery in the first trials of a practice block, but quickly dropped doing so during the practice phase. For instance, participants may experience the task as being a bit boring or find motor imagery too demanding. To test this hypothesis, we correlated mean inter-spacebar-intervals of the first four trials of the practice phase with the mean inter-spacebar intervals of the test phase. For the covert-practice condition, the Pearson product-moment correlation was not significant: *r* = .19, *p* = .29. For the overt-practice condition, the correlation was significant: *r* = .89, *p* < .001.

### Probe trial

Finally, we also considered performance on the probe trials of the practice phase. For all participants, RTs and error rates were available. For the analysis of the RTs, incorrect responses were discarded (6.25%). No additional outliers needed to be removed. The main effect of Practice Condition was significant, *F*(1,62) = 6.93, *MSe* = 77369, *p* < .05, $\eta_{p}^{2}$ = .07. RTs were faster following overt practice (*M* = 1,064 ms; *SE* = 49) then following covert practice (*M* = 1,217 ms; *SE* = 49). Error rates did not differ significantly as a function of Practice Condition, *F* < 1.

## Discussion

The aim of the current study was to test whether covert practice leads to automatic, and more specifically, unintentional behaviour. To this end, we used an item-specific priming paradigm and measured whether a response-congruency effect is present following a practice phase, in which participants imagined responding to a stimulus, without actually doing so. This effect was considered as a proxy for the storage of processing episodes during covert practice. Following a covert-practice phase, a response-congruency effect was observed on the RTs and error rates. We controlled whether the use of two sets of category-response instructions may in itself be sufficient to induce a response-congruency effect (e.g., Longman et al., 2019). New stimuli were added in the test phase, which were never responded to in the practice phase. The response-congruency effect for these new stimuli was not significant, thus suggesting that the impact of instructions was only minimal. New stimuli were also used to test whether the response-congruency effect induced by covert practice was mainly driven by a performance cost on incongruent trials or a performance benefit on congruent trials. The response-congruency effect was mainly the result of a performance cost, which suggests that this effect is based on the unintentional retrieval of a competing response. Taken together, our results suggest that during covert practice processing episodes are stored in memory, which are automatically retrieved when the relevant stimulus is presented. As such, we offer first evidence that covert practice is sufficient to trigger unintentional behaviour, which is a core characteristic of automaticity.

Whereas the results of the covert-practice condition were generally in line with the results of the overt-practice condition, we did observe that the performance benefit on congruent practised stimuli compared with congruent new stimuli was significant in the overt-practice condition but not in the covert-practice condition. However, our design was not optimal to draw strong conclusions about the difference between overt and covert practice. For instance, small differences in time parameters between both practice conditions were present. In the covert-practice condition, participants had a response limit of 4,000 ms to release the spacebar, imagine responding to the stimulus, and press the spacebar down again. In the overt-practice condition, participants had 2,000 ms to release the spacebar, 2,000 ms to press the response-key, and no response deadline was imposed for returning to the spacebar. Practice demands were thus not fully equated and future research should reevaluate the comparison between both conditions by taking these differences into consideration. In addition, a more proper comparison should also employ a larger sample.

The second question of the present study was whether covert practice was associated with motor imagery. To test this, we varied the physical distance between the release-key and the response-keys. The rationale of this manipulation is that covering a longer physical distance should also increase the time need to imagine making that response (e.g., Decety et al., 1989). In line with Theeuwes et al. (2018), we observed a longer time estimation when a larger physical distance needed to be covered. However, although this effect did not differ significantly between covert and overt responding, additional analyses indicated that this distance effect was mainly driven by the overt-practice condition. We also investigated the correlations between mean inter-spacebar-intervals in the test phase and mean inter-spacebar-intervals in the practice phase. This correlation was high in the overt-practice condition, but not in the covert-practice condition. Again this contrasts with our previous work (Scheil et al., 2020; Scheil & Liefooghe, 2018) in which we observed strong correlations between covert and overt responding. Taken together, we thus remain cautious in concluding that our results offer parsimonious support for the involvement of motor imagery during covert practice. On one hand, it is possible that our manipulation checks failed to clearly grasp the presence of motor imagery. For instance, in two experiments Theeuwes et al. (2018) found effects of physical distance on imagining time, but these authors used complex response sequences (entering a string of letters overtly or covertly, such as “E”-”P”-”T”). Furthermore, Scheil and colleagues observed strong correlations between overt and covert responding in designs which required participants to frequently switch between both response modes. These experimental parameters may have encouraged participants to lean more on kinesthetic components during covert practice.

Covert practice encompasses different processing components, and episodic storage may also have been mediated by visual imagery. Participants may have responded to the stimulus by visualising the keys or their finger pressing the key. Assuming that such visual strategy is perhaps less demanding for participants, it could account for the lack of outliers observed in the covert-practice condition. Finally, the role of inner speech also needs to be considered. Although the present study does not offer any direct evidence for the involvement of inner speech, our findings are in line with the results of Pfeuffer et al. (2017), who demonstrated that verbal messages induce automatic effects in the item-specific priming paradigm. Participants were presented with prime stimuli (e.g., pawn), which were accompanied with two verbal messages through a headphone: a category (e.g., non-mechanical) and a response (e.g., left). The stimuli needed to be classified in a later stage either as being mechanical versus non-mechanical or as being small versus large. In addition, the left-right response assignments to these categories varied. Probe stimuli could thus require a different categorization and/or response compared with the verbal messages presented together with the prime stimuli. Switching the category and/or the response between prime and probe triggered a performance cost in these studies (Pfeuffer et al., 2017), indicating that the verbal messages led to the formation of episodes in which stimulus, response, and category were related. Based on the study of Pfeuffer and colleagues, it could be hypothesised that when covertly practicing a task, participants rely on self-generated covert verbal messages, which relate a stimulus to a response (e.g., “car, left”). These covert verbal messages lead to traces in memory, which are automatically retrieved when they are irrelevant. As such, a response-congruency effect could also have been induced following covert practice via verbal messages.

It thus seems that although our experimental design incorporated manipulation checks to assess the role of motor imagery in inducing automatic effects, future research is needed. Individual differences with respect to participants’ ability in the technique of motor imagery could be considered (e.g., Zabicki et al., 2019) and/or the specific strategy used during covert practice (i.e., motor imagery, visual imagery, inner speech) explicitly instructed.

To conclude, our study indicates that covert practice can lead to (automatic) unintentional behaviour as indexed by a response-congruency effect. We propose that during covert practice, processing episodes are stored in long-term memory, which are automatically retrieved at a later stage. We acknowledge that future research will be needed to pinpoint the nature of the processes underlying covert practice, which are responsible for these effects (i.e., motor imagery, inner speech, visual imagery). However, it is clear that the present findings offer a new avenue for theories on automatic behaviour and research focusing on covert practice. On one hand, our findings strengthen the hypothesis that automaticity is not a sole function of overt practice. This conclusion is in line with previous demonstrations, which indicate that instructions (e.g., Liefooghe et al., 2012), verbal messages (e.g., Pfeuffer et al., 2017), or even derived learning (Liefooghe et al., 2020) can induce automaticity. As such, accounts of automaticity should be elaborated to accommodate such alternative pathways of learning (see Schmidt et al., 2020 for an example). On the other hand, the present results suggest that the benefit of imagery techniques in skill acquisition (see Driskell et al., 1994 for a meta-analysis) may be based on the formation of episodes in memory during practice, which over time lead to automatic behaviour.

However, we note that the overt and covert practices phase were not completely identical with respect to the response deadlines imposed to the participants. As such, this absence of a significant effect between both conditions could be reevaluated in future research.
